# Unilateral Angle Closure as the Initial Presentation of Multiple Myeloma Metastasis

**DOI:** 10.18502/jovr.v21.19965

**Published:** 2026-10-22

**Authors:** Thao Huong Nguyen, Truong Dang Minh Pham, Thanh Nghiep Trang, Ngoc Phuong Vy Ly, Hong Y Huynh

**Affiliations:** ^1^Ho Chi Minh City Eye Hospital, Xuan Hoa Ward, Ho Chi Minh City, Vietnam; ^2^Pham Ngoc Thach University of Medicine, Hoa Hung Ward, Ho Chi Minh City, Vietnam; ^3^Thao Huong Nguyen and Truong Dang Minh Pham contributed equally to this case report.

**Keywords:** Angle Closure, Metastasis, Multiple Myeloma

## Abstract

**Purpose:**

Ocular manifestations of multiple myeloma (MM) are rare but may affect all tissues of the eye. This report describes a patient with recurrent MM initially presenting with ocular hypertension due to angle closure, revealing extensive ocular, orbital, and systemic metastatic disease.

**Case Report:**

A 54-year-old female patient with previously treated MM presented with left eye pain, blurred vision, and an intraocular pressure of 38 mmHg. Examination revealed proptosis, restricted upward gaze, conjunctival injection with two salmon-patch lesions, and complete angle closure. Further findings included multiple ciliary body cysts, a massive choroidal mass, and retinal/choroidal detachments. She had pancytopenia. Magnetic resonance imaging confirmed recurrent MM with superior rectus muscle and spine infiltrations, and a large prevertebral lymph node mass. The patient responded well to antiglaucoma medications and was referred to her oncologist.

**Conclusion:**

MM can involve various ophthalmic structures. Patients with MM require regular examinations for early detection of ocular metastasis.

##  INTRODUCTION

Multiple myeloma (MM) is a malignant disease characterized by uncontrolled plasma cell proliferation, causing various systemic problems such as anemia, bone pain, and renal dysfunction.^[1]^

Ocular and orbital manifestations are rare yet diverse, potentially affecting all parts of the eye.^[1,2,3]^ Orbital involvement is uncommon but may indicate initial disease or recurrence.^[1,4]^ Secondary ocular hypertension caused by MM has been reported through various mechanisms.^[5]^ However, there are no documented cases of secondary ocular hypertension concurrent with MM infiltrations of the conjunctiva, ciliary body, choroid, and superior rectus muscle. This report describes a patient with MM who presented with ocular hypertension from angle closure, revealing extensive ocular, orbital, and systemic metastatic disease.

##  CASE PRESENTATION

A 54-year-old female with a history of MM presented with a 2-month history of blurred vision and pain in the left eye. Her MM, previously treated with bortezomib, lenalidomide, and dexamethasone, had been in complete remission for 1 year. Best-corrected visual acuity was counting fingers at 2.5 meters in the left eye (right eye: 20/40), and intraocular pressure (IOP) was significantly elevated at 38 mmHg in the left eye (right eye: 10 mmHg). Examination of the right eye was unremarkable, other than a narrow anterior chamber angle with visible trabecular meshwork upon indentation gonioscopy, and mild cataract. Examination of the left eye demonstrated proptosis, restricted upward gaze, conjunctival injection, vessel tortuosity, two distinct salmon patch lesions on the conjunctiva [Figure 1], and mild cataract. Gonioscopy showed complete angle closure [Figure 2]. Other ocular findings included retinal and peripheral choroidal detachments, subretinal fluid at the macula, and optic cup enlargement (cup-to-disc ratio of 0.5 compared with 0.3 in her right eye). Optical coherence tomography confirmed these signs and demonstrated increased thickness of the retinal nerve fiber layer [Figures 3 & 4].

B-scan ultrasonography revealed a hypoechoic mass above the left eye, temporal and inferior retinal detachments, and 360º peripheral choroidal effusion with heterogeneous lesions in the nasal suprachoroidal space [Figure 5]. Ultrasound biomicroscopy (UBM) showed hypoechoic masses in the positions corresponding to the conjunctival salmon patch lesions and multiple ciliary body cysts at various positions with a completely closed angle in every position [Figures 6A & 6B]. A massive and heterogeneous nasal choroidal mass was also confirmed on UBM. Notably, UBM of the fellow eye also demonstrated a narrow-angle configuration [Figure 6C].

Pancytopenia was observed (red blood cells: 3.96 x 10^6^/ μ L, platelets: 130 x 10^3^/ μ L, white blood cells: 3.64 x 10^3^/ μ L). Suspecting metastasis, we ordered whole-body magnetic resonance imaging (MRI), which revealed a well-defined intraocular tumor in the left globe, occupying half of the globe's volume, infiltrating the superior rectus muscle (24 × 13 mm), causing proptosis and inferior displacement of the globe [Figure 7]. The tumor exhibited low T1WI/T2WI signals with marked post-contrast enhancement. There were multiple tiny, scattered bone marrow lesions throughout the entire spine, and several nodules measuring 5-15 mm in the lumbar vertebral bodies, showing high signal on T2WI, low signal on T1WI, and strong post-contrast enhancement [Figure 8]. A homogeneously enhancing prevertebral lymph node mass (73 × 41 × 41 mm) was noted from L2 to L4, with a low T1WI signal, high T2WI signal, and intense post-contrast enhancement.

Although a biopsy of the conjunctival mass was recommended, the patient declined due to financial constraints. The clinical and imaging findings were highly suggestive of metastatic MM invading the lymph node, spine, conjunctiva, ciliary body, choroid, and superior rectus muscle, resulting in secondary angle-closure glaucoma. Topical antiglaucoma medications (brimonidine, timolol, and brinzolamide) and systemic acetazolamide (500 mg twice daily) were initiated, resulting in a prompt response. The IOP in the OS normalized to 14 mmHg within 5 days of initiating treatment, remaining stable and controlled following the discontinuation of the 5-day course of oral therapy. At this point, follow-up gonioscopy revealed a persistently narrow angle, and UBM showed unchanged ciliary body cysts and choroidal effusion. Following the discovery of metastatic disease, the patient was referred to her oncologist for further treatment. However, the patient declined further investigations, and treatment and was subsequently lost to follow-up.

**Figure 1:**
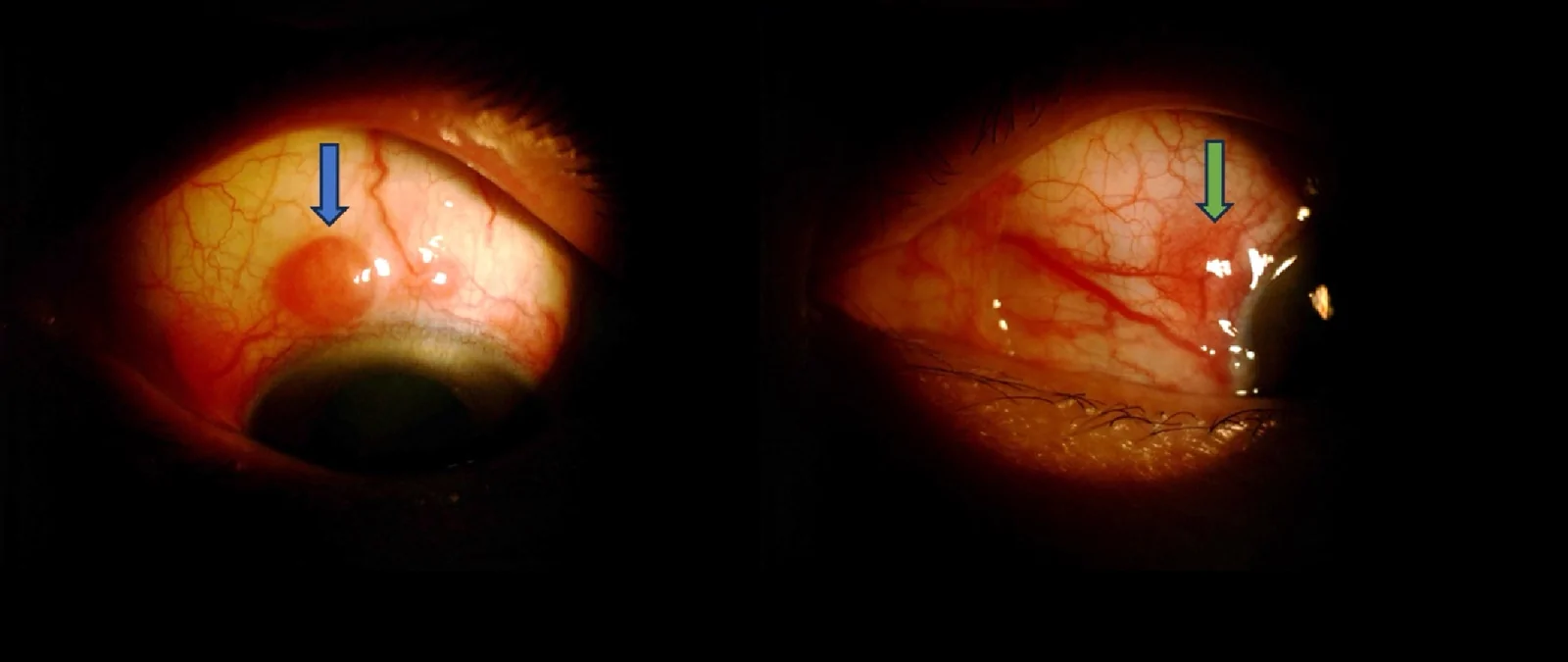
Two distinct conjunctival salmon patch lesions. One was seen at the 12 o'clock position (blue arrow) and another at the 9 o'clock position (green arrow).

**Figure 2:**
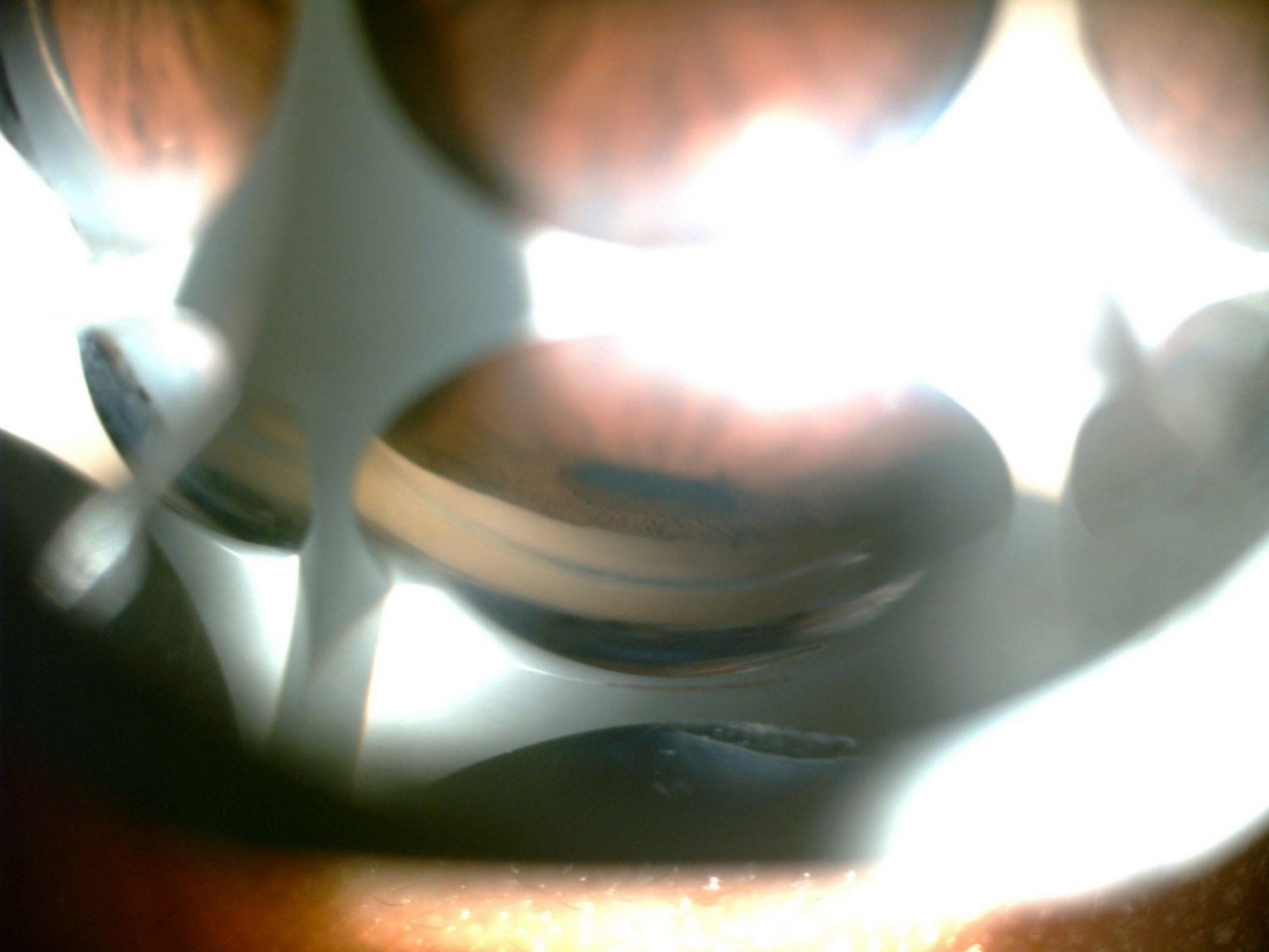
Gonioscopy with indentation of the left eye showed complete angle closure with no visualization of anterior chamber structures.

**Figure 3:**
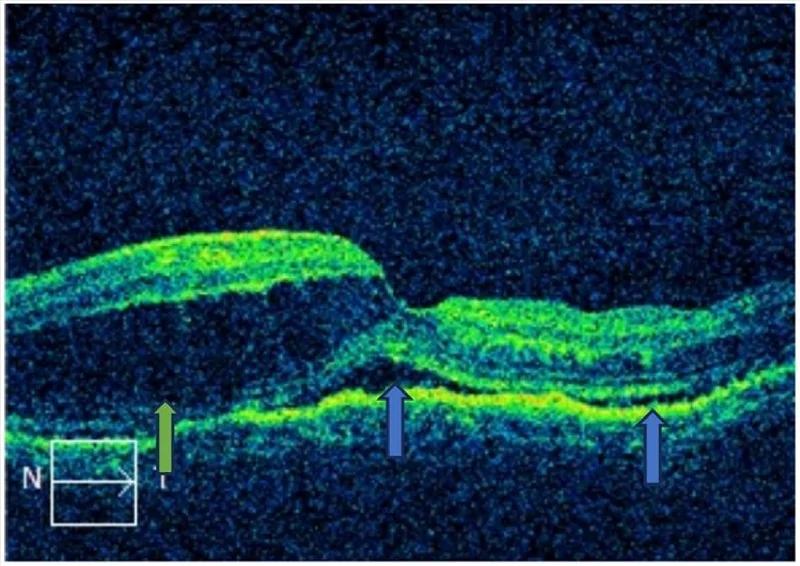
Macular optical coherence tomography scan of the left eye. Subretinal fluid (blue arrows) and intraretinal fluid (green arrow).

**Figure 4:**
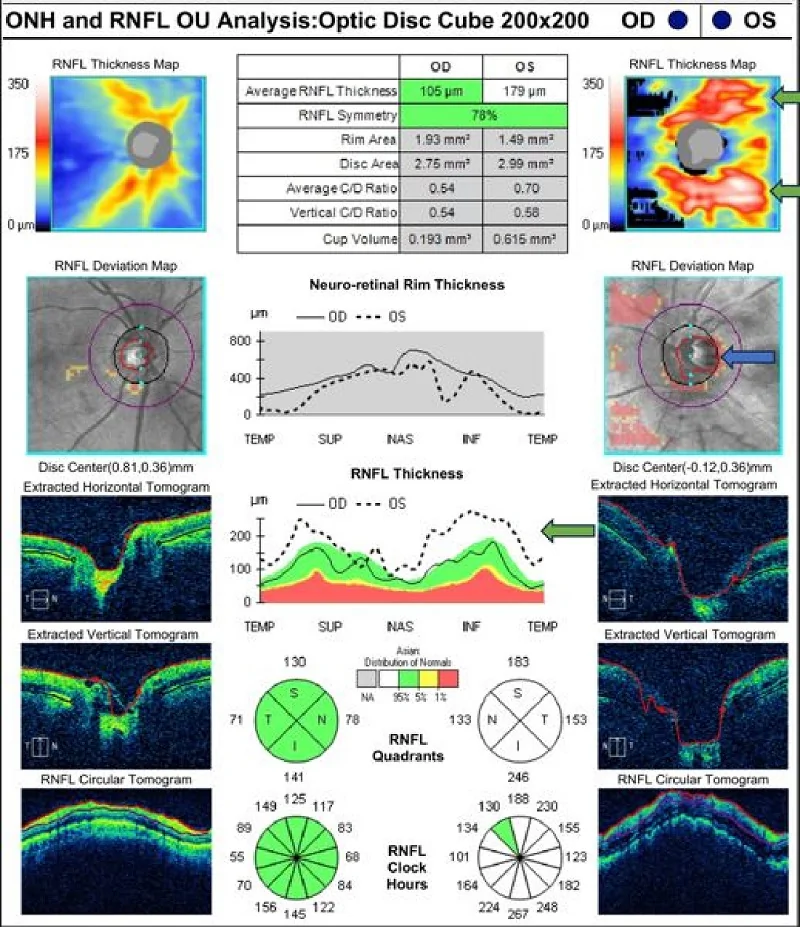
Optic nerve head and retinal nerve fiber layer analysis by optical coherence tomography. An increased optic cup (blue arrow) and increased thickness of the retinal nerve fiber layer are noted (green arrows) in the left eye.

**Figure 5:**
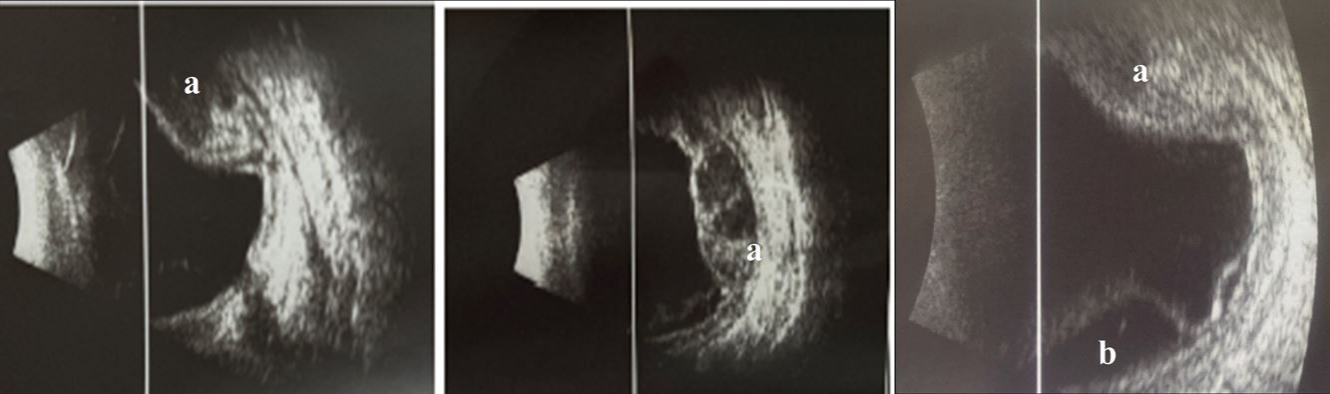
B-scan ultrasonography of the left eye demonstrating heterogenous lesions in the nasal suprachoroidal space (a) and temporal retinal detachment (b).

**Figure 6:**
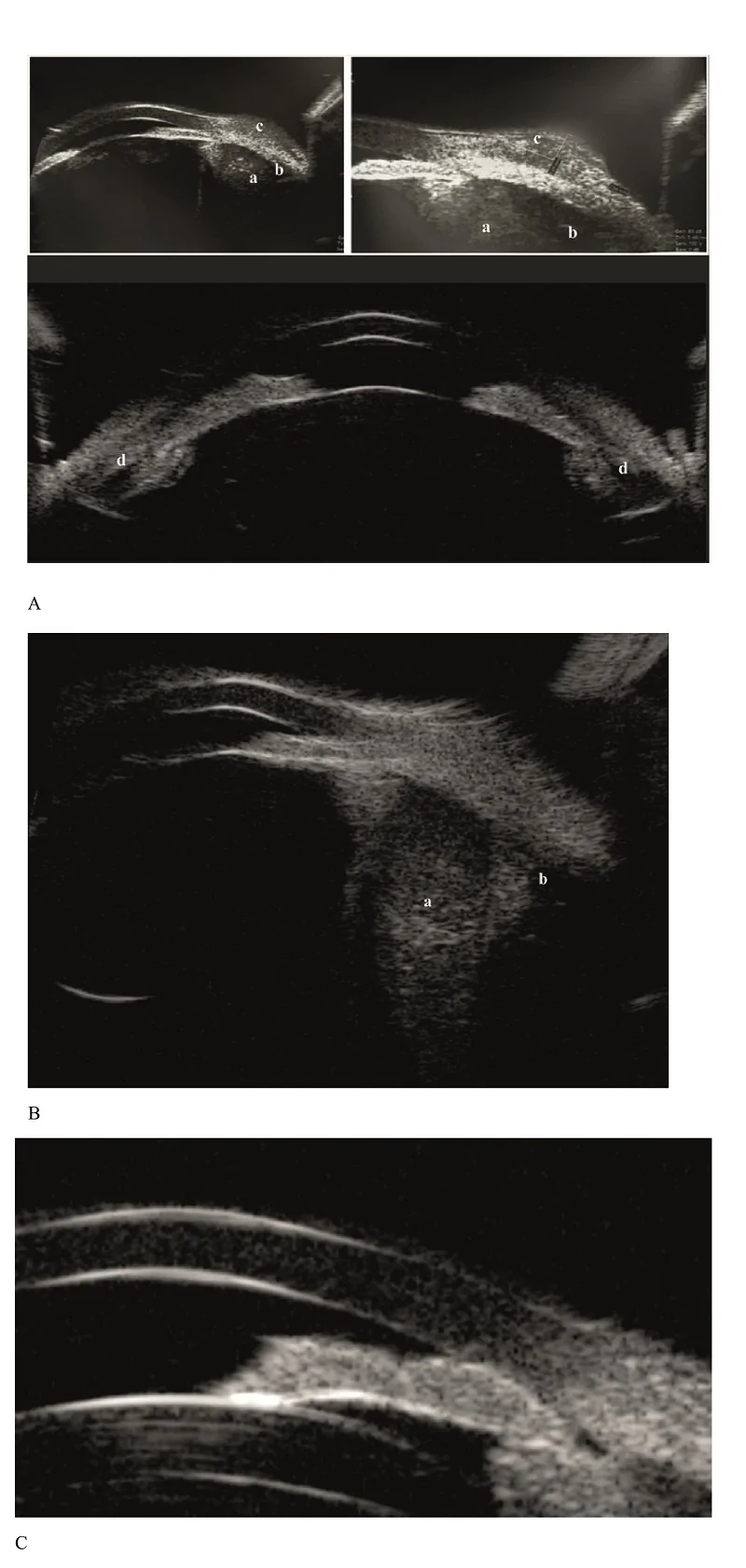
(A) Ultrasound biomicroscopy of the left eye demonstrated a massive and heterogenous nasal suprachoroidal mass (a), choroidal effusion (b), hypoechoic conjunctival masses (c), and multiple ciliary cysts (d). (B) Ultrasound biomicroscopy of the left eye also demonstrated a heterogenous nasal suprachoroidal mass (a) and choroidal effusion (b). (C) Ultrasound biomicroscopy of the right eye revealing a narrowed anterior chamber angle.

**Figure 7:**
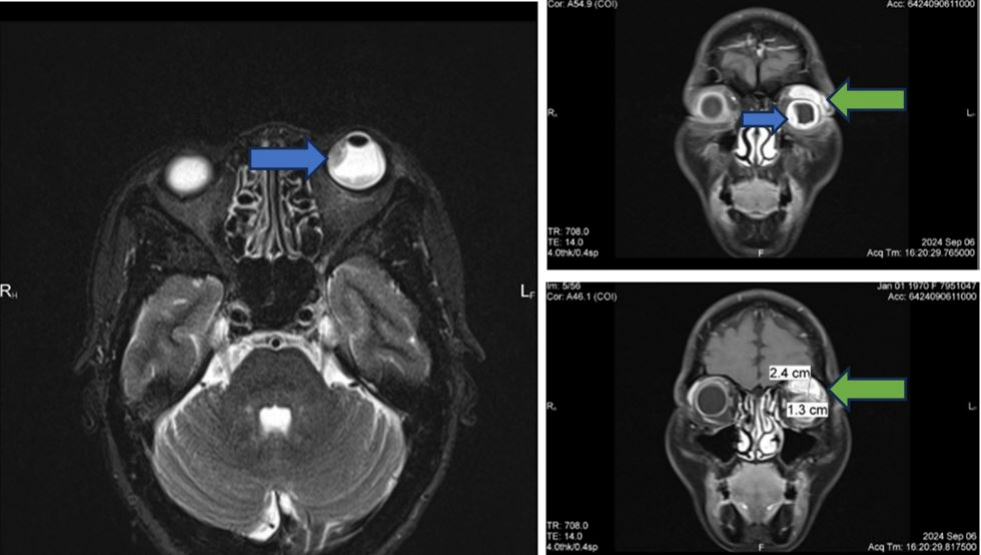
Superior rectus infiltration (green arrows) and intraocular tumor (blue arrows) were observed on the magnetic resonance imaging: axial plane (left), and coronal plane (upper and lower right).

**Figure 8:**
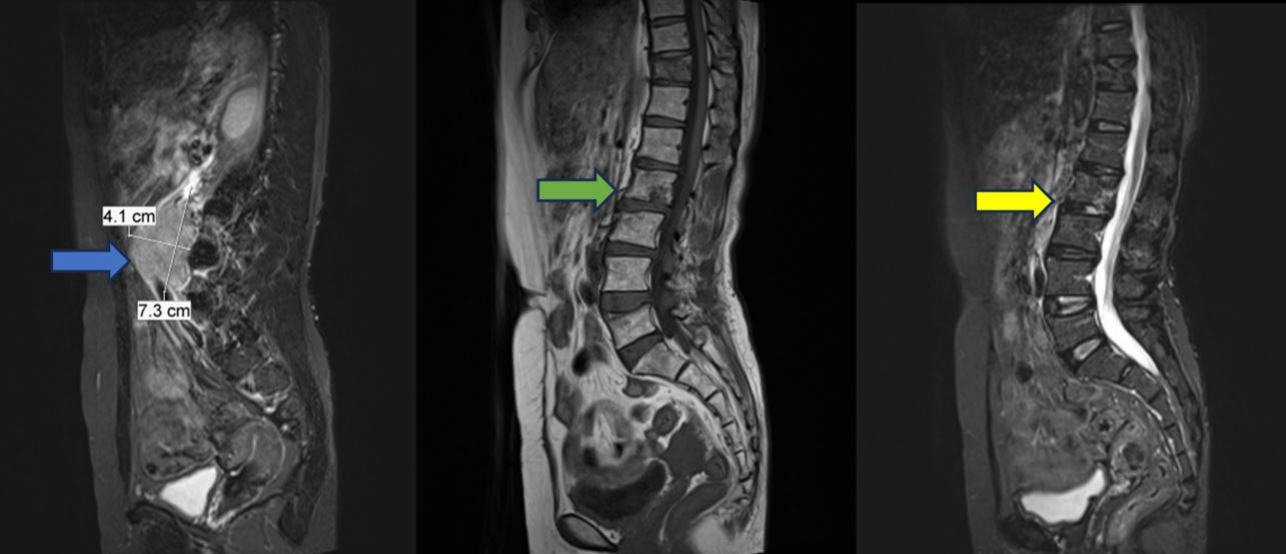
Whole-body magnetic resonance imaging (MRI) revealed a massive prevertebral lymph node caused by metastasis (blue arrow, left image) and nodules in the lumbar vertebral bodies on T1WI (green arrow, middle image) and on T2WI (yellow arrow, right image).

##  DISCUSSION

Conjunctival involvement in MM is exceedingly rare.^[6]^ To our knowledge, previous literature reports only solitary conjunctival lesions as a secondary manifestation of MM.^[7,8,9,10,11,12]^ However, our case is unique because it presented with two distinct conjunctival lesions alongside massive prevertebral lymph node metastasis found on whole-body MRI. Existing studies suggest that any conjunctival involvement may signal a more aggressive disease course and a poorer prognosis.^[6,12,13]^ Case reports have shown that multiple extramedullary plasmacytomas involving the choroid and conjunctiva are associated with an aggressive disease and a poorer prognosis.^[9,13]^ These reports have suggested that even a solitary conjunctival lesion may be a marker of more aggressive disease. Our case, featuring multifocal conjunctival involvement alongside multi-organ metastasis, supports this notion and raises the possibility that multiple conjunctival lesions could indicate an even higher degree of systemic disease involvement. Given these potential implications for prognosis and treatment, routine ophthalmological screening for conjunctival lesions in patients with MM is warranted to facilitate timely intervention and optimize patient management.

Ocular hypertension in metastatic MM is a rare but significant finding. The underlying pathophysiology is diverse, involving various mechanisms. Gerecke et al highlighted the potential for secondary glaucoma to develop as a consequence of myeloma therapy.^[14]^ They noted that bilateral angle closure occurred in a patient following the administration of daratumumab, a monoclonal antibody used to treat relapsed MM. Lee et al reported a similar case of bilateral angle closure following daratumumab infusion.^[15]^ Baker et al described a case of angle-closure glaucoma potentially related to multiple factors, including relaxation of the zonular fibers, anterior displacement of the lens and iris base by ciliary body cysts, and recurring iridocyclitis.^[16]^ Chin et al detailed a case where an MM of the iris and ciliary body led to secondary angle-closure glaucoma, highlighting the potential for direct tumor involvement to cause IOP elevation.^[2]^ Other reported mechanisms include iris and anterior chamber infiltration,^[17]^ orbital processes causing indentation of the eyeball,^[18]^ obstruction of trabecular meshwork,^[5,19]^ and neovascularization.^[12,20]^ In our case, the angle closure causing ocular hypertension could be attributed to ciliary body cysts and eyeball indentation from superior rectus infiltration, consistent with the reports of Baker^[16]^ and Kottler,^[18]^ respectively. However, our case demonstrated additional contributing factors. Unlike Baker's patient, who had no choroidal involvement, our patient presented with both choroidal effusion and tumor infiltration, which further contributed to the hypertension through increased intraocular volume confirmed by UBM and MRI. Similarly, while Kottler described globe indentation secondary to an orbital inferonasal mass, our imaging revealed that the indentation resulted from direct infiltration of the superior rectus muscle. Mohamed et al reported a case of unilateral multiple extramedullary plasmacytoma, in which choroidal detachment and infiltration were found with a concurrence of mildly elevated IOP of 23 mmHg.^[9]^ However, the ocular hypertension observed in this case report was not as high as in our case, which was 38 mmHg during the patient's initial presentation, nor were there other mechanisms in action as in our case. To our knowledge, the concurrence of ciliary body cysts, superior rectus infiltration, and choroidal manifestations has not been previously reported. Given the anatomical predisposition to angle closure, evidenced by the narrow anterior chamber angle of the fellow eye, the affected eye developed ocular hypertension following MM infiltration via the described mechanisms. Considering the extension of choroidal effusion and ciliary body cysts, the posterior pushing mechanism appeared to be the primary cause of ocular hypertension. These findings suggest that multiple concurrent mechanisms can contribute to ocular hypertension in metastatic MM, and the severity may reflect the extent of multifocal ocular involvement. Ultimately, the diversity of mechanisms underlying elevated IOP requires thorough ophthalmological evaluation in patients with MM presenting with ocular hypertension to determine the underlying cause and guide appropriate management as soon as possible, effectively preserving functional vision in these patients.

While orbital tumors ( < 1%) and extraocular muscle infiltration (5.9%) are individually rare manifestations of MM,^[4,18]^ their simultaneous occurrence with multifocal conjunctival and intraocular metastasis has not been documented. The uniqueness of this case resides in the cumulative rarity of these concurrent signs. To our knowledge, no prior report describes a “pan-ocular” involvement—affecting the conjunctiva, ciliary body, choroid, and superior rectus muscle—concurrently with systemic lymphatic and spinal metastasis. Therefore, metastatic disease should be considered in the differential diagnosis of unusual ocular presentations, especially when there is a history of malignancy.

Despite the compelling clinical and multimodal imaging evidence of widespread ocular, orbital, and systemic involvement in the context of MM, the diagnosis was not confirmed by biopsy. This limitation necessitated a diagnosis of exclusion, whereby inflammatory, infectious, or autoimmune processes mimicking metastasis could not be definitively ruled out. However, the strong temporal and clinical correlation with the patient's known malignancy and concurrent pancytopenia—further supported by characteristic bone lesions on MRI, a diagnostic criterion for MM per the International Myeloma Working Group^[21]^—and the absence of inflammatory, infectious, or autoimmune history strongly suggest metastatic MM as the most likely diagnosis.

This case highlights the possibility of simultaneous widespread metastatic involvements affecting the conjunctiva, ciliary body, choroid, and superior rectus muscle. Patients with a history of MM and new ocular symptoms or elevated IOP require a complete ophthalmic examination to check for disease progression. The clinical presentations of this case provide valuable insights that may assist in the diagnosis and management of patients with MM.

##  AI Use Disclosure

During the preparation of this work, the authors used Gemini 2.5 Flash to improve the language and check the spelling and grammar of the article, without altering its content. After using this tool, the authors reviewed and edited the content as needed and take full responsibility for the content of the published article.

##  Financial Support and Sponsorship

None.

##  Conflicts of Interest

None.
